# Vendor evaluation platform for acquisition of medical equipment based on multi-criteria decision-making approach

**DOI:** 10.1038/s41598-023-38902-3

**Published:** 2023-08-07

**Authors:** Neven Saleh, Mohamed N. Gaber, Mohamed A. Eldosoky, Ahmed M. Soliman

**Affiliations:** 1 Biomedical Engineering Department, Higher Institute of Engineering, Shorouk Academy, Cairo, Egypt; 2 Military Technical College, Cairo, Egypt; 3 Biomedical Engineering Department, Faculty of Engineering, Helwan University, Cairo, Egypt

**Keywords:** Health care, Engineering, Mathematics and computing

## Abstract

The purchase of medical equipment is a critical issue that should be planned properly. The selection of the most appropriate vendor impacts time, effort, and expenses. Therefore, the challenge is to strike a balance between the available budget and the required equipment. The study aims to select the best vendor for supplying medical equipment based on Emergency Care Research Institute (ECRI) standards. The multi-criteria decision-making approach has been adopted through three methods; Multi-Objective Optimization by Ratio Analysis (MOORA), Simple Additive Weighting (SAW), and Technique for Order Preference by Similarity to Ideal Solution (TOPSIS). The criteria of selection are divided into general, technical, and financial. The criteria are weighted using three methods: CRITIC, entropy, and expert judgment. The Vendor Evaluation Program for Medical Equipment (VEPME) is designed to automatically select the best vendor. Medical imaging equipment is selected to test the program by four modalities: X-ray equipment, CT, MRI, and ultrasound. The best scenario was given by the entropy-TOPSIS. As a result, this methodology was adopted by the program. The results demonstrate the robustness of the proposed methodology by comparing the VEPME output to expert judgment.

## Introduction

Medical technology is at the core of healthcare delivery systems. The key role of medical technology should be underlined in the Health Technology Management (HTM) process. Indeed, the HTM process encompasses many phases, including acquisition, which is a decision-making-based phase^1^. According to the World Health Organization, a percentage of 50 to 70 percent of medical devices in low- and middle-income countries are in poor usage due to a lack of an appropriate management policy^2^. One challenge is to optimize the costs without compromising the quality of healthcare. To respond effectively, the acquisition of medical equipment must be shaped properly in terms of planning, selection, and resource allocation.

The environment in which decisions about suppliers and their ability to deliver are made is complex, with high levels of ambiguity and uncertainty, competing stakeholder values, and complicated relationships as a result of multiple competing objectives^3^. Also, complications arise in identifying appropriate and relevant criteria and assigning appropriate weights, all of which are likely to vary as a function of many factors. In this way, a lot of time, effort, and expenses are spent evaluating and selecting medical equipment. However, it must be carefully chosen to ensure that it functions properly.

The study addresses the acquisition process concerning the objective selection of medical technology based on standardization. Therefore, a set of evaluation criteria as well as standard guidelines for specifications must be considered. The Emergency Care Research Institute (ECRI) is an international independent organization concerned with healthcare practices. In this context, the ECRI guidelines for technical specifications of medical equipment are adopted as a standard reference^4, 5^.

The Multi-Criteria Decision-Making (MCDM) approach is a decision-making methodology that maps alternatives to be selected against a set of evaluation criteria. According to the weights of the criteria, preference is given to the most appropriate alternatives^6^. In this way, the MCDM is employed for resolving complex decision-making issues, particularly with conflict criteria. In literature, several MCDM tools have been utilized in various applications, such as Analytical Hierarchy Process (AHP), Multi-Objective Optimization by Ratio Analysis (MOORA), Simple Additive Weighting (SAW), Technique for Order Preference by Similarity to Ideal Solution (TOPSIS), and elimination and choice expressing reality (ELECTRE)^6–8^. Besides, criteria weighting reflects the importance of the criteria; therefore, how to calculate those weights is a key problem in the MCDM^9^. There are three main categories for criteria weighting methods, namely, objective-based methods, subjective-based methods, and integrated methods^10^. In objective-based methods, the weights are given based upon the information presented by the alternatives, meanwhile, subjective-based methods extract the weights based upon an expert’s preference. The integrated methods combine both methods. Among objective-based methods, the entropy method and the Criterion Importance via Inter Criteria Correlation (CRITIC) method are commonly adopted^11, 12^.

The study aims to develop an evaluation policy for medical equipment vendors using the SAW, TOPSIS, and MOORA. Further, criteria weighting is calculated using entropy, CRITIC, and experts. Moreover, an automated evaluation platform is designed to manage the process conveniently. The contributions of the study are summarized as follows: (i) solve the problem of selecting the best vendor by using the MCDM approach; (ii) design a novel platform to automatically select the best vendor; (iii) use ECRI standards for benchmarking; and (iv) save effort, time, and cost in medical equipment acquisition.

The structure of this article is organized as follows. Section "Related works" discusses the works that are related to the problem at hand. The methodology, including the preliminaries of the MCDM methods are given in Section "Methodology". Section "Results" presents the results of the implementation. The discussion of the study is provided in Section "Discussion". Finally, Section "Conclusions" concludes the work and gives guidelines to the future work.

## Related works

Optimizing the acquisition of medical technology is a critical aspect that should be customized carefully. Unbiased evaluation of tenders is one feature of a good medical technology procurement. From a vendor selection perspective, making a judgment involves compromising a level of ambiguity and complexity^13^. The significance of evaluation criteria for vendor selection has been examined under different circumstances. Together, cost, quality, and conveyance execution were distinguished as the main rules. Comparative studies that were conducted by Cardozo^14^ have declared the role of the quality level of a product or a service in vendor’s selection. The resilience of the contractor is another criterion highlighted by Alper et al.^15^ to select the best supplier for providing an integrated logistic information system. The problem of vendor selection was solved by the AHP and a heuristic technique to optimize the alternatives^16^.

For medical equipment, a multi-criteria fuzzy-based system has been developed to select the most appropriate vendor for supplying medical equipment. According to the study, the most important criteria are the company, product quality, and after-sales service^17^. The selection of mobile X-ray equipment has been evaluated using value engineering and the TOPSIS method^18^. Failure mode and effect analysis was combined with fuzzy logic to improve the purchasing process in public hospitals. Another study used the quality function deployment and fuzzy logic approach to prioritize the acquisition of medical equipment^19^. According to our best knowledge, the studies conducted to automatically evaluate and select the most appropriate vendor for medical equipment referring to standards are rarely considered in the literature.

## Methodology

Tender evaluation identifies the most appropriate device, which is the one with the highest criteria and the lowest cost. Therefore, the methodology comprises two stages. First, the MCDM approach is used to select the best vendor based on a set of criteria considering ECRI standards. Second, an automated platform is developed to evaluate and rank all the vendors. The SAW, TOPSIS, MOORA, entropy, and CRITIC methods are explained as follows.

### Preliminaries

#### Simple additive weighting

The Simple Additive Weighting (SAW) method is developed based on a weighted summation of the performance of each alternative over the selected criteria^20^. The principle is a superior alternative gets the highest score, while the worst alternative gets the lowest score^21^. Let *x*_*ij*_* represent* score of alternative *i* (i = 1,…,*n*), based on criterion *j* (*j* = 1,…,*m*), and *w*_*j*_ represent the weight score for criterion *j*. The normalization process takes place for each *x*_*ij*_ by calculating the normalized value *r*_*ij*_ based upon the type of criterion; beneficial or non-beneficial. In the case of beneficial criteria, a good criterion must be maximized, meanwhile, for non-beneficial criteria, it must be minimized. This rule is generalized for all MCDM methods. Using (3), the score *S*_*i*_ is assigned to each alternative.

1 $$r_{{{\text{ij}}}} = x_{{{\text{ij}}}} /Max_{{{\text{ij}}}} \left( {{\text{beneficial}}} \right)$$

2 $${\text{r}}_{{{\text{ij}}}} = Max_{{{\text{ij}}}} /x_{{{\text{ij}}}} \left( {{\text{non}} - {\text{beneficial}}} \right)$$

3 $$Si{ } = \mathop \sum \limits_{i = 1}^{n} {\text{w}}_{{\text{j }}} {\text{r}}_{{{\text{ij}}}}$$

#### Technique for order preference by similarity to ideal solution

The Technique for Order Preference by Similarity to Ideal Solution (TOPSIS) was initiated for the first time in 1981 by Hwang and Yoon. The idea of the TOPSIS method is to determine the ideal solution. In this case, we have two ideal solutions; the positive ideal solution that maximizes the benefits of the alternatives, and the negative ideal solution that minimizes the benefits. Subsequently, the closeness of each alternative to both ideal solutions is measured. The best solution is the one with the shortest distance to the ideal positive solution and at the same time the longest distance to the ideal negative solution^21, 22^. Implementation of the TOPSIS method is carried out in six steps, as described below.

*Step 1: *Formulation of the decision matrix by listing the alternatives A_i_, (i = 1,…, n) against the criteria C_j_, (j = 1,…,m) to find the element *x*_*ij*_*.*

*Step 2: *Decision matrix normalization by applying Eq. (4) to calculate the normalized value *r*_*ij*_ for each element.

4 $${{\varvec{r}}}_{{\varvec{i}}{\varvec{j}}}=\frac{{{\varvec{X}}}_{{\varvec{i}}{\varvec{j}}}}{\sqrt{\sum_{{\varvec{i}}=1}^{{\varvec{n}}}{{{\varvec{X}}}_{{\varvec{i}}{\varvec{j}}}}^{2}}},\boldsymbol{ }\boldsymbol{ }\boldsymbol{ }\boldsymbol{ }{\varvec{i}}=1,\boldsymbol{ }2,\boldsymbol{ }\dots .,{\varvec{n}}\boldsymbol{ }\boldsymbol{ }\boldsymbol{ }\boldsymbol{ }{\varvec{j}}=\mathrm{1,2},\dots ..,{\varvec{m}}$$

*Step 3:* Establishing the weighted normalized matrix *V*_*ij*_ by multiplying the weight *w* of the j^th^ criterion by the normalized value *r*_*ij*_

5 $$V_{ij} = w_{j} r_{ij}$$

*Step 4:* Determining the positive ideal solution A^+^ and the negative ideal solution A^-^ using Eqs. (6) and (7) respectively.

6 $${A}^{+}=\left\{{{V}_{1}}^{*}, \dots \dots .{{V}_{m}}^{*}\right\}=\left\{\left({max}_{j} {v}_{ij} j\in {\Omega }_{b}\right)|\left({min}_{j} {v}_{ij} j\in {\Omega }_{c}\right)\right\}$$

7 $${A}^{-}=\left\{{{V}_{1}}^{-}, \dots \dots .{{V}_{m}}^{*-}\right\}= \left\{(min {v}_{ij} j\in {\Omega }_{b})|({max}_{j} {v}_{ij} j\in {\Omega }_{c})\right\}$$

*Step 5:* Calculation of the Euclidean distances.

Euclidean distance is a separation of each alternative from the positive ideal solution and negative ideal solution as shown in Eqs. (8) and (9) respectively.

8 $${{D}_{i}}^{+}=\sqrt{\sum_{j=1}^{m}{({v}_{ij}-{{v}_{j}}^{*})}^{2} ,}i=1,\dots \dots .,n$$

9 $${{D}_{i}}^{-}=\sqrt{\sum_{j=1}^{m}{({v}_{ij}-{{v}_{j}}^{-})}^{2} },i=1,\dots \dots .,n$$

*Step 6:* Relative closeness computation.

To rank the alternatives, the relative closeness of each alternative to the ideal solution is determined using Eq. (10). Taking into account, RC ranges from 0 to 1. The ranking is given in descending order according to the highest value of ${RC}_{i}$.

10 $${RC}_{i}=\frac{{{D}_{i}}^{-}}{{{D}_{i}}^{*}+{{D}_{i}}^{-}} ,i=1,\dots \dots .,n$$

#### Multi-objective optimization by ratio analysis

The multi-Objective Optimization by Ratio Analysis (MOORA) method was initiated by Brauers in 2004^21^. It is one of the compensatory methods in which desirable and undesirable criteria are used. It is employed in several applications, such as supplier selection^23, 24^. The steps of the MOORA method are described as shown below.

**Step 1**, **step 2**, and **step 3** are the same as described in the TOPSIS method. **Step 4**: Determine the reference point as a classification of the benefits criteria and cost criteria as shown in Eqs. (11), (12) respectively.

11 $$\sum\nolimits_{{j = 1}}^{g} {{\text{wj}}\;{\text{rij}}}$$

12 $$\sum\nolimits_{{j = g + 1}}^{n} {{\text{wj}}\;{\text{rij}}}$$

where *g* represents the number of benefit criteria and *n-g* is the number of cost criteria.

**Step 5**: Determine the overall performance index for each alternative B _j_

13 $$B_{j} = \mathop \sum \limits_{j = 1}^{g} {\text{w}}_{{\text{j}}} {\text{ r}}_{{{\text{ij}}}} - \mathop \sum \limits_{j = g + 1}^{n} {\text{w}}_{{\text{j}}} {\text{ r}}_{{{\text{ij}}}}$$

**Step 6**: The ranking is given in descending order according to the highest value of B^-^_j_.

#### Entropy method

Basically, to determine the best alternative, it is required to measure all alternatives against a set of criteria. As a result, the primary goal of the entropy method is to calculate the weight of criteria based on the uncertainty information assigned to each criterion. The index reflects the degree of criterion dispersion^25^. The method is carried out in three steps, as shown below.

*Step 1:* Normalization of each element of the decision matrix as in Eq. (4).

*Step 2:* Calculation of the entropy value (*e*_*j*_) of each normalized element as shown in Eq. (14).

14 $${{\varvec{e}}}_{{\varvec{j}}}=-\frac{\left[{\sum }_{j=1}^{m}{r}_{ij}\mathrm{ln}{r}_{ij}\right]}{\mathrm{ln}n} ; i=\mathrm{1,2},..\dots ,n ; j=\mathrm{1,2},..\dots ,m,$$

*Step 3:* Calculating the relative weight (Wj) of each criterion as presented in Eq. (15).

15 $${{\varvec{w}}}_{{\varvec{j}}}=\frac{1- {e}_{j}}{{\sum }_{j=1}^{m}1- {e}_{j}} j=\mathrm{1,2},..\dots ,m,$$

#### CRITIC method

The Criterion Importance Through Intercriteria Correlation (CRITIC) method is one of the most common methods for calculating criteria weight. It was presented for the first time by Diakoulaki in 1995^26^. The CRITIC method reflects correlation among the data in addition to the strength of the data contrast. The following steps describe how the criteria weights are calculated by the CRITIC.

*Step 1:* Normalization of each *x*_*ij*_ in the decision matrix using the maximum value and minimum value as shown in Eq. (16).

16 $${r}_{ij}=\frac{{x}_{ij}-{x}_{j}^{min}}{{x}_{j}^{max}-{x}_{j}^{min}}$$

*Step 2:* Calculation of correlation coefficient Cj as in Eq. (17)

17 $${c}_{j}={\sigma }_{j}{\sum }_{j=1}^{m}\left(1-{r}_{ij}\right)$$

*Step 3: *Calculation of the weight as in Eq. (18)

18 $${w}_{j}=\frac{{c}_{j}}{{\sum }_{i=1}^{m}{c}_{i}}$$

### Application of the MCDM for vendor selection

To benchmark vendors, we need to list them against medical equipment specifications. At this point, we proposed classifying the specifications into general, technical, and financial. General specifications include warranties, training requirements, and certifications. Technical specifications are assigned based on ECRI standards, whereas financial specifications are all expenses related to the cost of the device. A score index is generated for each criterion separately to rank the overall quotation. The SAW, TOPSIS, and MOORA methods are applied as described above. The weights of the three categories of the specifications are given based on entropy, CRITIC, and expert judgment.

For expert judgment, four states are estimated as follows: If the choice specifications do not meet the minimum requirements, the option will be removed from the evaluation procedures. If the specifications do not match the basic specifications, they will not score. If the specifications meet the standard requirements, it will achieve total scoring. If the chosen specification outperforms the standard requirements, it will receive total credit for that specification. Furthermore, the option would receive a higher score in the final percentile of the total score for that specification. A score is given for each category separately to conclude the overall score index and indicate a preference for the best one.

### Vendor evaluation program for medical equipment (VEPME)

**Figure 1:**
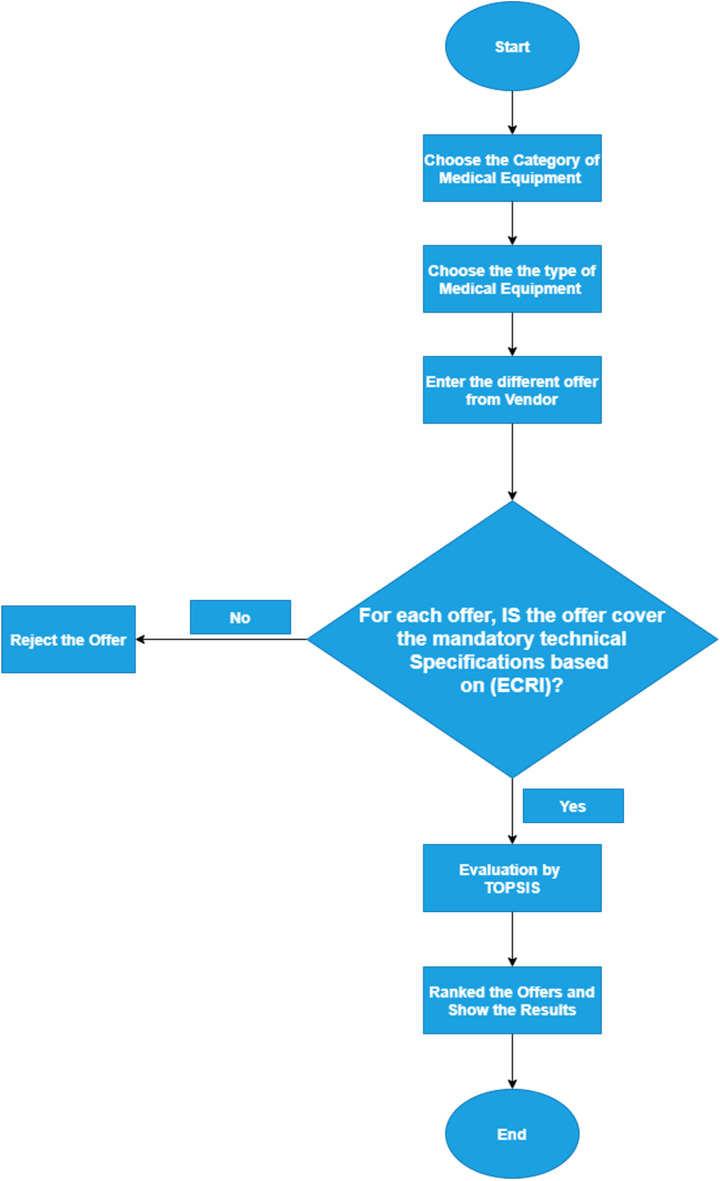
The flowchart of the VEPME program demonstrating the sequence of work flow.

The study proposes the simulation of expertise in decision making by developing a software program named Vendor Evaluation Program for Medical Equipment (VEPME). The VEPME uses an internet browsing service as a customer, and it is characterized by its ease of interface and lack of time commitment. The frame was designed using JavaScript as a basic programming language because it worked on a domestic hosting service. Initially, the user selects the type of medical equipment, and then the specifications are assigned in terms of general, technical, and financial conditions. All quotations are evaluated in the VEPME based on the entropy-TOPSIS method. The flowchart of the suggested VEPME is depicted in Fig. 1.

### Ethical approval and consent to participate

This article does not contain any studies with human participants performed by any of the authors.

## Results

The study presents a framework for evaluating the vendors of radiology devices to select the optimal one. The best offer is chosen based on its general, technical, and financial characteristics. To demonstrate the results of the developed models, assume we have five vendors named A, B, D, E, and F for purchasing digital X-ray equipment. The general and technical specifications of the quotations are evaluated based on the expertise described in Section "Application of the MCDM for vendor selection". Additionally, the prices for medical equipment are given as shown in Table 1.

**Table 1:** A comparison among five vendors according to prices, technical scores, and general scores.

Vendor	Price (EGB)	Technical scores	General scores
A	5,000,000	90	86
B	7,000,000	95	90
D	7,500,000	95	100
E	5,500,000	91	93
F	5,500,000	84	90

### Results of the MCDM approach

**Figure 2:**
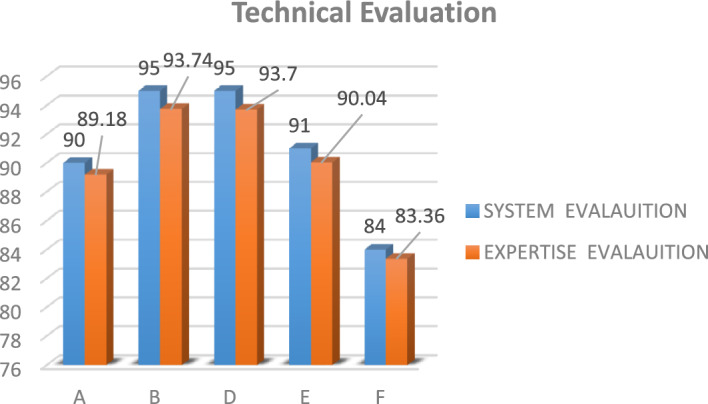
A comparison for vendors’ technical evaluation between the experts and the proposed system.

**Figure 3:**
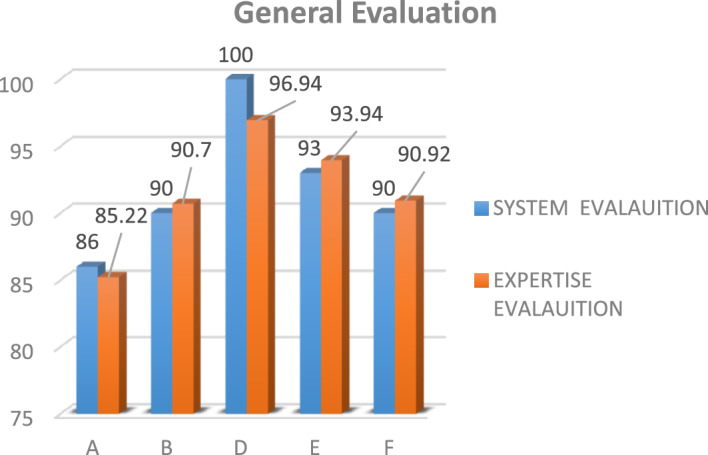
A comparison for vendors’ general evaluation between the experts and the proposed system.

The MCDM approach is adopted for the underlining problem by applying SAW, TOPSIS, and MOORA for ranking in addition to three methods for criterion weighting. Table 2 shows the results of applying CRITIC, entropy, and expertise to weight criteria. The results of applying the MCDM methods are depicted in Table 3. To examine the best method for weight calculation, a correlation coefficient is determined for each pair of the MCDM methods, as shown in Table 4. The system has been validated by 50 experts with an average of (15 ± 3.07) years of experience. Area of experience is a list of all activities related to the HTM, specifically radiology medical devices. A survey has been conducted through a questionnaire to assess the proposed five vendors. Comparisons of technical evaluation and general evaluation are introduced in Figs. 2 and 3, respectively.

**Table 2:** Results of calculating the weights of criteria using CRITIC, entropy, and expertise methods.

	CRITIC	Entropy	Expertise
Financial weight	0.46	0.845343	0.3
Technical weight	0.28	0.067928	0.5
General weight	0.26	0.086729	0.2

**Table 3:** Ranking results of applying MOORA, TOPSIS, and SAW on the five vendors.

	MOORA	TOPSIS	SAW	
A	1	1	1	CRITIC
B	4	4	5	
D	5	5	4	
E	2	2	2	
F	3	3	3	
A	1	1	1	Entropy
B	4	4	4	
D	5	5	5	
E	2	2	2	
F	3	3	3	
A	1	1	1	Expertise
B	4	4	5	
D	5	5	3	
E	2	2	2	
F	3	3	4

**Table 4:** The correlation coefficient among the adopted methods with respect to the CRITIC, entropy, and the expertise.

	CRITIC	Entropy	Expertise
MOORA-TOPSIS	1	1	1
MOORA-SAW	0.9	1	0.7
TOPSIS-SAW	0.9	1	0.7

### Results of the VEPME program

**Figure 4:**
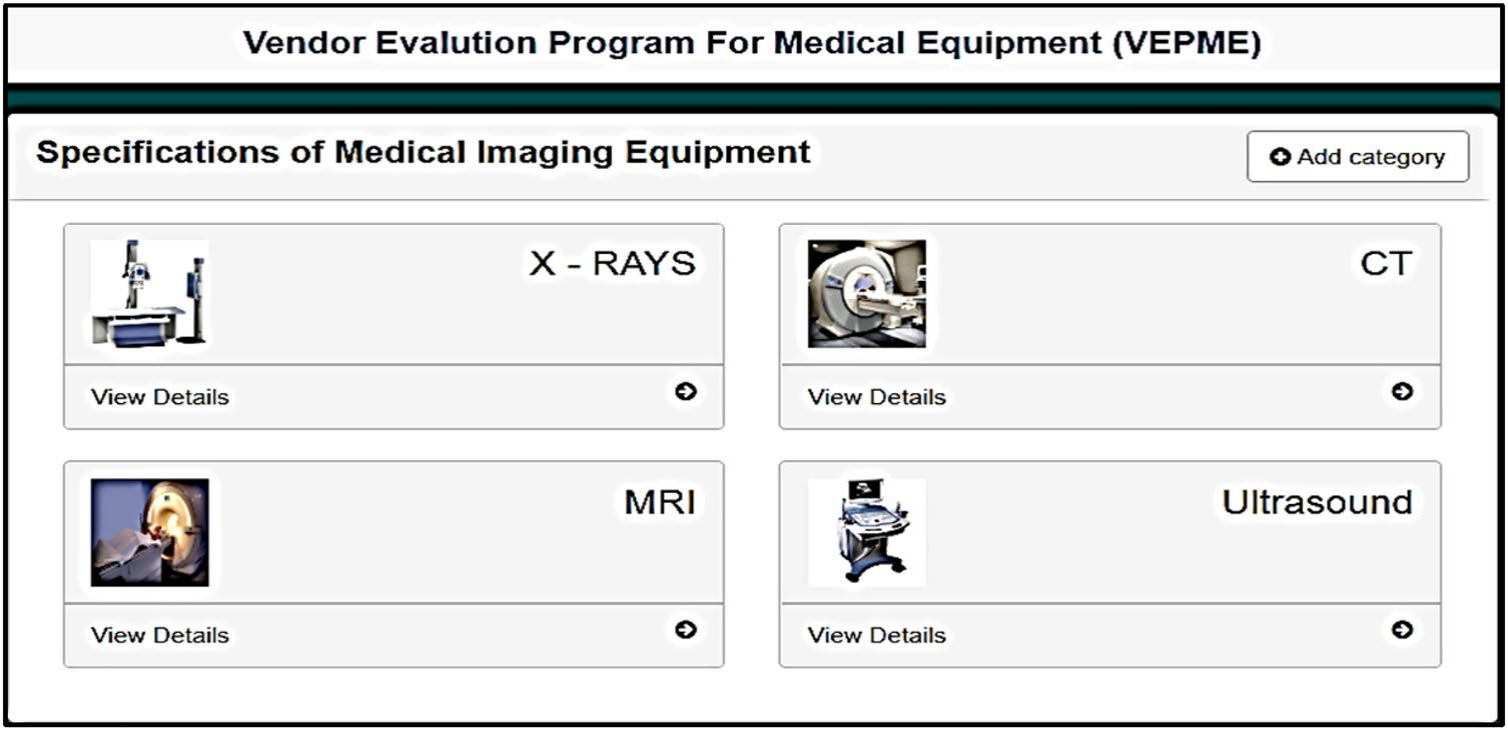
The general GUI for the VEPME program for selecting a piece of medical imaging equipment.

**Figure 5:**
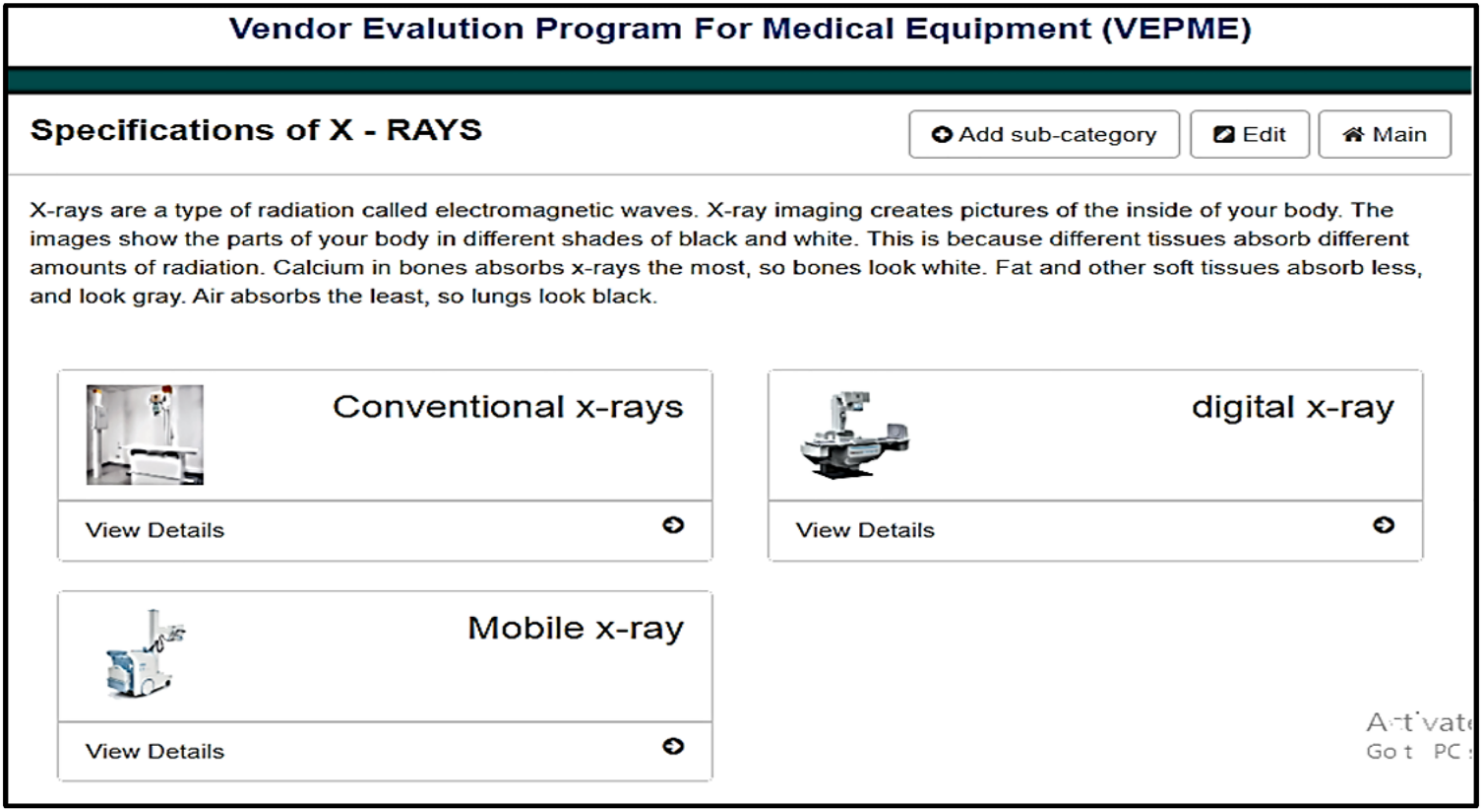
The window for selecting sub-categories of the X-ray equipment according to the VEPME program.

**Figure 6:**
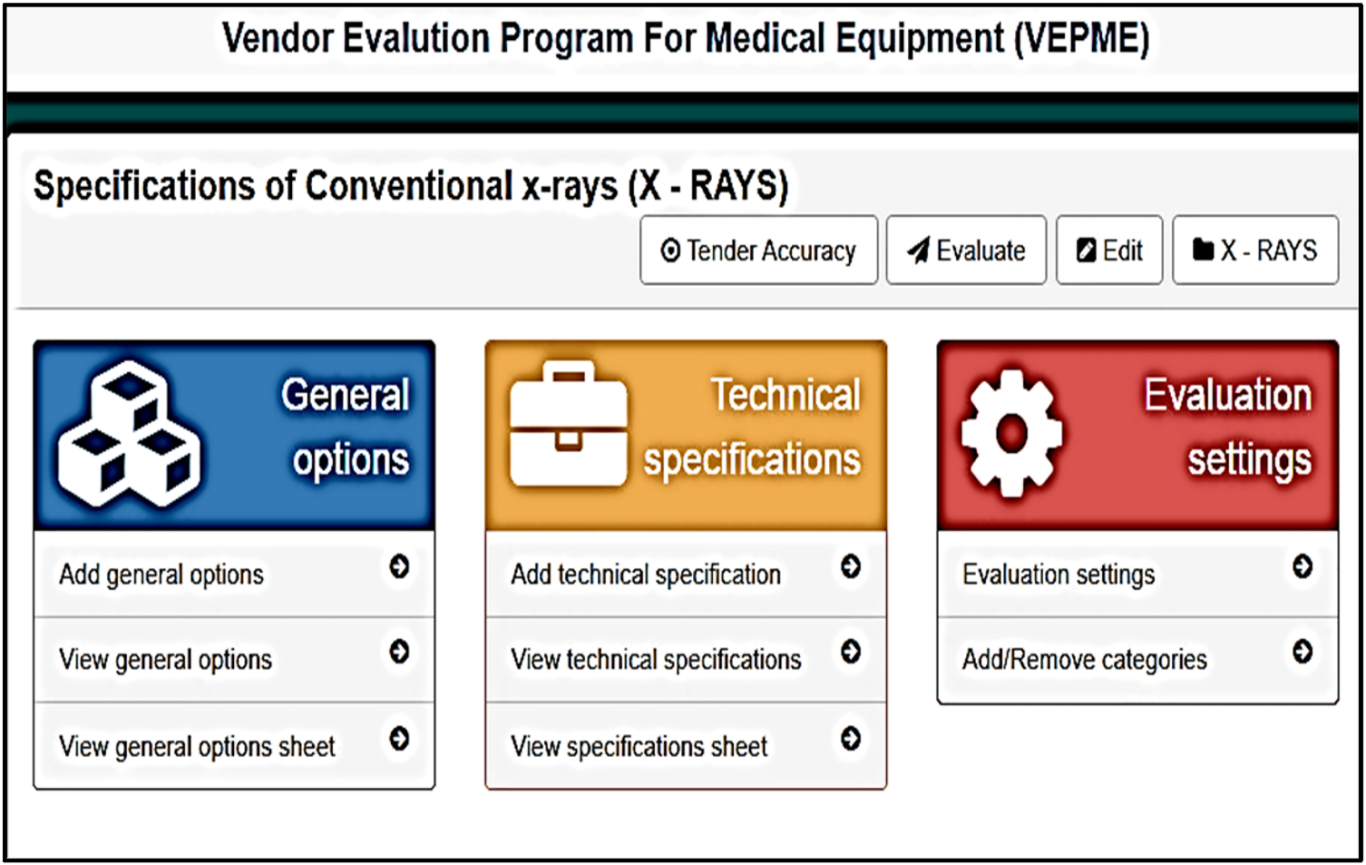
An example of the VEPME window for general, technical, and financial specifications of conventional X-ray equipment.

**Figure 7:**
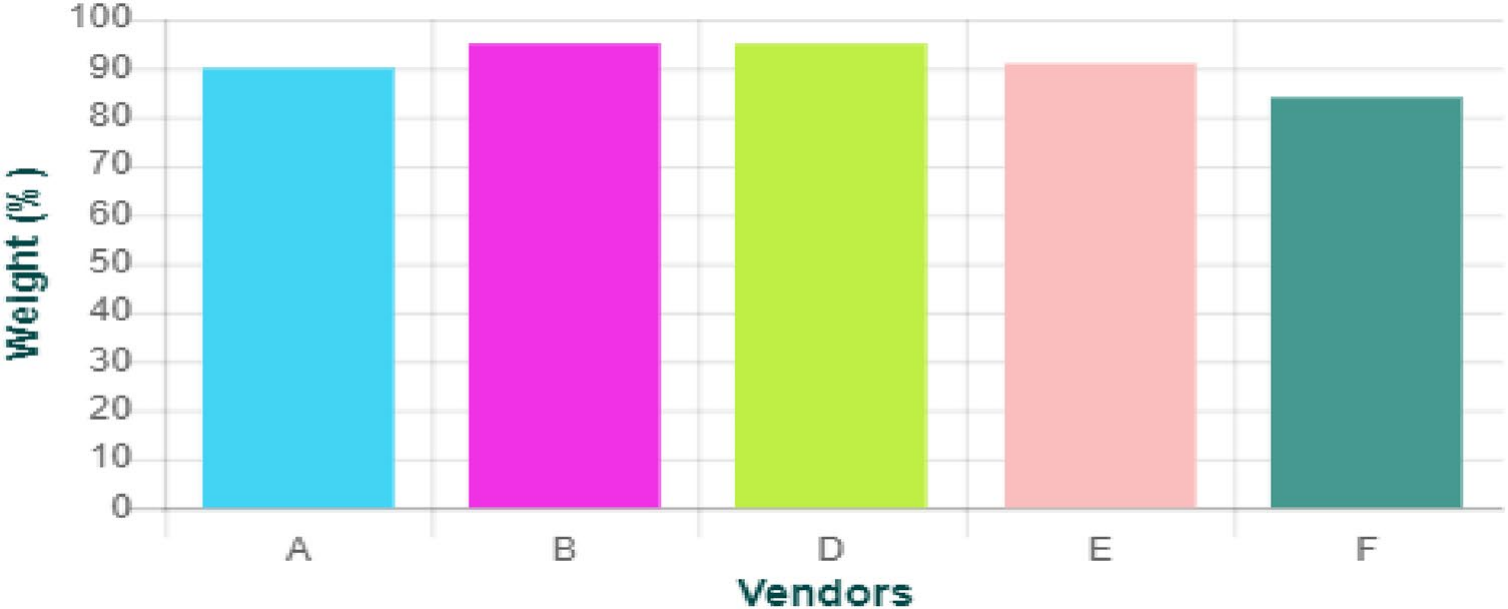
A bar chart demonstrates evaluation results of the vendors for purchasing conventional X-ray.

**Figure 8:**
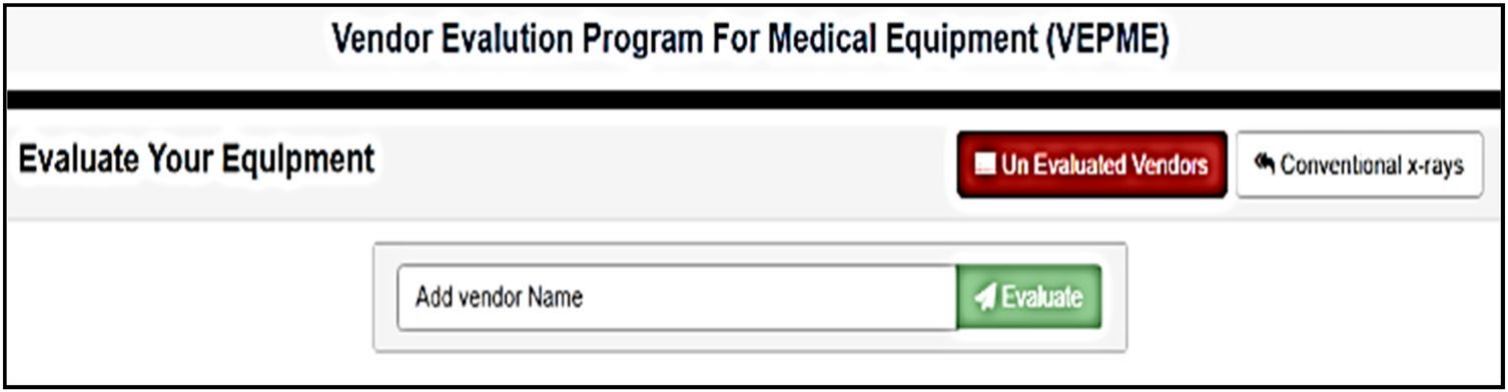
The window of adding vendor in the VEPME program.

The VEPME is a software program that acts as a web application. It is characterized by ease of interface, flexibility, and dependability. The main GUI window of the program is designed for imaging medical equipment; X-ray, CT, MRI, and ultrasound, as depicted in Fig. 4. Besides, a new category can be added as the field of “add category” is depicted in Fig. 4. The user selects the category and sub-categories of the imaging modality as shown in Fig. 5. Also, a new sub-category can be added as shown in Fig. 5. For instance, the X-ray equipment is subdivided into digital, conventional, and mobile equipment. An example of selecting a vendor for conventional X-ray is given in Fig. 6. Taking this into account, the VEPME refuses any quotation that does not fulfil the mandatory requirements of ECRI standards for the technical specifications. All vendors are assessed and ranked by the program based on the entropy-TOPSIS method. By selecting the sub-category, all competitive vendors are benchmarked with respect to the general, technical, and financial specifications. The VEPME presents the results of comparison in report form and many forms of chart, such as line, bar, and pie, as demonstrated in Fig. 7. For clarification, assume we need to purchase an ultrasound device, we are required to follow the incoming steps. First, select the "ultrasound category" as indicated in Fig. 4. Second, select the required sub-category. Third, add the general specifications, the technical specifications according to the ECRI standards, and the evaluation settings as shown in Fig. 6. Fourth, add the vendor name as shown in Fig. 8. Fifth, for each technical criterion, a score is given, leading to a final score for the technical evaluation. The same is conducted for general and financial evaluations. Steps 4 and 5 are repeated for each vendor. The entropy-TOPSIS method is used to rank all vendors within the VEPME. Finally, a summary report is generated to demonstrate the evaluation of the vendors and their ranking.

## Discussion

An automated vendor evaluation platform was established to choose the best vendor for imaging equipment. Four modalities have been tested, X-ray, CT, MRI, and ultrasound. Three methods of the MCDM have been employed: TOPSIS, MOORA, and SAW. All vendors are evaluated based on standardization referred to as ECRI standards. Scores are generated for each vendor with respect to technical, general, and financial specifications. The vendors that are not aligned with the mandatory specifications are rejected automatically.

The ranking of the vendors is varied according to the adopted method for criteria weight and the MCDM method. For example, vendor "B" ranked fourth in MOORA and TOPSIS for both methods the CRITIC and expertise. Meanwhile, it was ranked fifth using the SAW for the same methods of weighing the criteria. Also, it has been noted that the ranking results of the MOORA and the TOPSIS are identical, whereas the ranking of the SAW is quite different. Although various methods are used for criterion weight, it seems they are similar. The similarity is due to the convergence of raw data values. Moreover, the correlation coefficient results demonstrate the consistency of the entropy method over the other methods.

Technical and general evaluations of both the experts and the system revealed no significant differences. It means the system proves its ability to evaluate the vendors based on the given criteria. The evaluation charts, in their various forms, make judging the presented quotations easier. Additionally, the VEPME outlines steps for applying the TOPSIS method to assess the vendors. The main advantage of the program is its dynamics, because it can modify the results of the evaluation depending on the criteria. The GUIs of the program are characterized by ease of interface. The user can interact with the VEPME without prior training.

Comparing the study to relevant works reveals its strength. Saleh et al.^19^ have presented a conceptual priority index for the purchase to rank the required equipment. As a result, no evaluation of the vendor is provided. Although, the problem of vendor selection was studied by Chakraborty et al.^16^, automation of the solution is not included. In the work^22^, only the expert-TOPSIS method was used in evaluating the vendor. Three MCDM methods with three criteria weighting methods were used in our proposed methodology. Moreover, an automated program was developed for implementation. Unlike the evaluation of vendor performance in the purchasing process of medical equipment, other aspects of vendor evaluation were presented. The performance of the vendor was evaluated by Ouda et al.^27^ in terms of providing timely maintenance and spare parts. Additionally, the impact of vendor performance on the availability of medical equipment in emergency department was discussed in the study^28^. The limitations of the study are that only imaging equipment was chosen for the testing. Four modalities were selected for application. Additionally, one case study has been conducted for the purchase of conventional X-ray equipment. More types of medical equipment can be investigated. Furthermore, the VEPME was designed based on the TOPSIS, but further methods can be adopted. The VEPME program was operated by a local host server, instead, an internet server could be used.

## Conclusions

In medical equipment management, many challenges face the decision makers. One of them is to strike a balance between the available budget for the purchase and the standard specifications. The goal of the study is to aid hospitals in purchasing the most appropriate medical equipment based on an assessment of the specifications. To assess vendors, three terms—namely, technical specifications, general specifications, and financial specifications are examined. The MCDM approach is adopted by applying SAW, TOPSIS, and MOORA. Also, three methods were used for weighting criteria; CRITIC, entropy, and expertise. The best scenario is the entropy-TOPSIS method. Therefore, applying many methods of the MCDM approach has proven its consistency.

The ECRI standard is one of the most respected worldwide standards for benchmarking. Using the standards ensures an unbiased evaluation. An automated platform called the VEPME was designed for implementation. By using the VEPME program, all vendors that fulfil the mandatory specifications are assessed and ranked numerically and visually. The program is characterized by its ease of interface and dynamics. Automatic assessment of vendors impacts time, effort, and expenditures. Future work extends to using more methods of the MCDM and more types of medical equipment. Moreover, the VEPME could be a mobile application.

## Data Availability

The datasets used and/or analyzed during the current study are available from the corresponding author on reasonable request.

## Abbreviations

AHP: Analytical hierarchy process
CRITIC: Criterion importance via inter criteria correlation
ECRI: Emergency care research institute
ELECTRE: Elimination and Choice Expressing Reality
HTM: Healthcare technology management
MCDM: Multi-criteria decision-making
MOORA: Multi-objective optimization by ratio analysis
SAW: Simple additive weighting
TOPSIS: Technique for order preference by similarity to ideal solution
VEPME: Vendor evaluation program for medical equipment
